# A life course study of genetic and environmental influences on sustainable working life

**DOI:** 10.1371/journal.pone.0317675

**Published:** 2025-02-25

**Authors:** Annina Ropponen, Jacob Bergström, Jurgita Narusyte, Pia Svedberg

**Affiliations:** 1 Division of Insurance Medicine, Department of Clinical Neuroscience, Karolinska Institutet, Stockholm, Sweden; 2 Finnish Institute of Occupational Health, Helsinki, Finland; Shenzhen Baoan Women's and Children's Hospital, CHINA

## Abstract

Genetics plays a role in short- and long-term sustainable working life (i.e., not having interruptions due to sickness absences (SA), disability pensions (DP), or unemployment), but the life course effects are not known. Thus, we aimed to investigate the age-specific genetic and environmental influences on sustainable working life from young adulthood until old-age pension. We used classical twin modeling based on the genetic relatedness of mono- and dizygotic twins in a longitudinal design. The final sample (n =  49 372) of Swedish same-sex twins with known zygosity born between 1929 and 1990 (52.8% women) with detailed national register data of employment, SA, DP, unemployment, old-age pension, emigration, and death. Genetic influences for sustainable working life were 54% at ages 18–27 years, 59% at 28–37 years, 37% at 38–47 years, 69% at 48–57 years, and 34% at 58–65 years. We observed genetic influences transferred from 18–27 years to 28–37 years and to 38–47 years explaining 28% and 17% of the variance, respectively, from 28–37 years to 38–47 years 60%, and from 48–57 years to 58–65 years 47%. Unique environmental influences were 57–72% in five age groups. Age group-specific common environment explained 63% of the variance for 18–27 years, 27% for 28–37 years, 12% for 48–57 years, and 25% for 58–65 years (none for 38–47 years). From age 48–57 years, the common environment explained 24% of the variance among those aged 58–67. To conclude, stability and change in genetic effects are important for a sustainable working life across the life course. The unique environmental effects were important for the middle age groups between 28 and 57 years. Thus, society and workplaces should support health and well-being to promote a sustainable working life.

## Introduction

Sustainable working life has previously been defined as “not having or having very little work incapacity, that is sickness absence (SA) or disability pension (DP), or other interruptions such as unemployment” based on Swedish register data [1–3]. From a societal perspective, the rates of interruptions in participation to paid work due to SA/DP are high and have also increased over time in developed countries despite improved health [4, 5], which is a result of, e.g., an increase in life expectancy [4–6]. On the other hand, societal demands of raising the retirement age and also increasing the proportion of the total population in paid work require a sustainable working life [7]. Furthermore, the known association between higher age and SA/DP [8, 9], and that unemployment in early adulthood predicts SA/DP [10], also highlights the need for a life course perspective. Therefore, the fit between work and individual characteristics including genetics and circumstances related to the environment during the life course should be emphasized [11]. A recent study based on Swedish twins about the role of genetic and environmental factors in sustainable working life for a two year or a long time (20 years) indicated that both moderate unique and common environmental effects, and to a lower extent genetic effects contributed to individual differences in sustainable working life using a period prevalence estimate [2]. However, more detailed knowledge is needed to better understand the factors influencing the sustainability of work over the life course.

Stability and change in genetic and environmental influences on sustainable working life have not been studied to the best of our knowledge. Instead, earlier studies have focused on modeling genetic and environmental effects for work incapacity, i.e., SA and/or DP[12–16]. However, a sustainable working life is also known to carry a genetic component[2], why assumptions exist that an investigation accounting for stability and changes in genetic influences across the follow-up might be merited. Understanding the influence of genetics in sustainable working life across life courses could promote timing and possibilities of interventions or other actions for workability and employment.

Therefore, we aimed to investigate the age-specific genetic and environmental influences on sustainable working life from young adulthood until old-age pension. We aimed to test age-cohort differences in the type, magnitude, and stability of these influences across the life span.

## Materials and methods

The study sample is based on the Swedish Twin project Of Disability pension and Sickness absence (STODS) including the twins identified in the Swedish Twin Registry (STR). Twins born between 1925 and 1990, i.e., 119 907 twin individuals were included. Zygosity (monozygotic, MZ or dizygotic, DZ) for the STODS same-sex twins has been determined by STR based on survey questions about childhood resemblance. The method has 98% accuracy when validated against serological and microsatellite markers [17, 18].

The whole cohort with all data (see below) included 108 275 twin individuals. The final sample (n =  49 372 was restricted to only same-sex twin pairs with known zygosity; they were born between 1929 and 1990 (52.8% women). Table 1 reports the complete concordant and discordant pairs for sustainable working life across zygosity. The final sample was categorized into 10-year age intervals from 18–27 years to 58–65 years where we cut the upper limit into 65 years as that is the most common age for old-age pension for the period studied in Sweden, following the procedure reported before [3].

**Table 1:** Number of concordant and discordant twin pairs in the final sample (n =  49,378) for sustainable working life by 10-year age intervals.

			Age intervals for sustainable working life										
			Concordant^**^						Discordant				
**N pairs** ^*^	**18**–**27**		**28**–**37**	**38**–**47**	**48**–**57**	**58**–**65**	**18**–**27**		**28**–**37**	**38**–**47**	**48**–**57**	**58**–**65**
**MZ**	11,378	893		3,314	4,422	3,854	1,889	1,084		1,853	1,774	1,986	1,904
**DZ**	13,308	631		2,832	4,480	4,651	2,539	952		2,040	2,631	3,413	3,314

* At cohort entry the number of pairs equals the final sample of 49 372 individuals via formula (2*11378 + 2*13308).

** Concordant pairs regarding unsustainable working life, 18–27: MZ 2,786; DZ 1,869, 28–27: MZ 1,077; DZ 816, 38–47: MZ 879; DZ 780, 48–57: MZ 1,027; DZ 1,245, 58–65: MZ 1,631; DZ 2,307

### National register data

For this study, apart from zygosity we utilized national register data only and analyzed the data between the 1^st^ of January 1994 and 31^st^ of December 2021. Information regarding SA and DP was from the Swedish Social Insurance Agency, and employment (being in paid work) or unemployment was from the Longitudinal Integration Database for Health Insurance and Labor Market Studies (LISA), Statistics Sweden (SCB) [19] until 2021. The date of death came from the Cause of Death Register from the National Board of Health and Welfare, and emigration and old-age pension data were from the LISA, SCB. Death, emigration, old-age pensions, and reaching 65 years were censored during the follow-up.

### Sustainable working life

The degree of sustainable working life was estimated using the main labor market status in each year of follow-up based on the definitions used before [20]: SA/DP ( > 180 days with SA or DP benefits from the Social Insurance Agency); unemployment ( > 180 days with unemployment benefits); old-age pension (more than half of yearly income from the old-age pension) [21, 22]; or employment (i.e., in paid work and did not fulfill the criteria SA/DP, unemployment, or old-age pension). The sustainable working life was estimated as a binary variable, i.e., these statuses were coded for each year being in paid work and did not fulfill the criteria SA/DP, unemployment, or old-age pension (as a proxy for employment) =  “1” and all other statuses “0”.

The power calculations assuming a heritability (h^2^) of 20% for sustainable working life (at 8% prevalence for the oldest age group) would require slightly more than 6,000 twin pairs (1/3 MZ and 2/3 DZ) to reject the hypothesis. The power of this test, at the 0.05 significance level with 1 degree of freedom is 0.98. Assuming the heritability equals 30% a sample size of approximately 3 000 twin pairs is needed. Our sample provides enough sample size to study sustainable working life across age groups.

### Statistical analyses

The data was organized into age intervals (18–27, 28–37, 38–47, 48–57, and 58–65 years) at baseline. We assessed the within-pair similarity (i.e., to measure if one twin has a sustainable working life, what is the probability that the other twin has that) in sustainable working life across age groups by calculating within-pair correlations for MZ vs. DZ, and men vs. women to measure concordance within twin pairs [23, 24] for the first description of the importance of genetic and environmental influences.

We applied a Cholesky decomposition model, in which phenotypic variances are decomposed into genetic (A), common environmental (C), and unique environmental (E) components for each observed age interval of sustainable working life. Then the sustainable working life was ordered in time, the Cholesky ACE model decomposition was thereafter interpreted as a longitudinal model [12,25]. That means that each genetic and environmental component was assumed to influence observations later in time, but not earlier. The model was fitted with Full Information Maximum Likelihood as an estimation procedure for raw data in umx within R (see https://cran.r-project.org/web/packages/umx/umx.pdf). The model was first fitted men and women separately, but due to the power issues, sexes were combined into the same model accounting for sex.

We compared alternative models for genetic and environmental components (i.e., ADE, AE, CE, and E models) using Akaike’s Information Criteria (AIC), and Bayesian Information Criteria (BIC) seeking the best data fitting and the most parsimonious and best fitting model to the data.

The study protocol was designed and performed according to the principles of the Helsinki Declaration. The ethical vetting was performed and approved by the Regional Ethical Review Board of Stockholm, Sweden (Dnr: 2007/524-31; 2010/1346-32-5; 2017/128-32). For this project, the Regional Ethical Review Board of Stockholm stated that the consent to participate was not applicable in these types of large register studies. Authors only had access to pseudonymized data.

## Results

**Fig 1:**
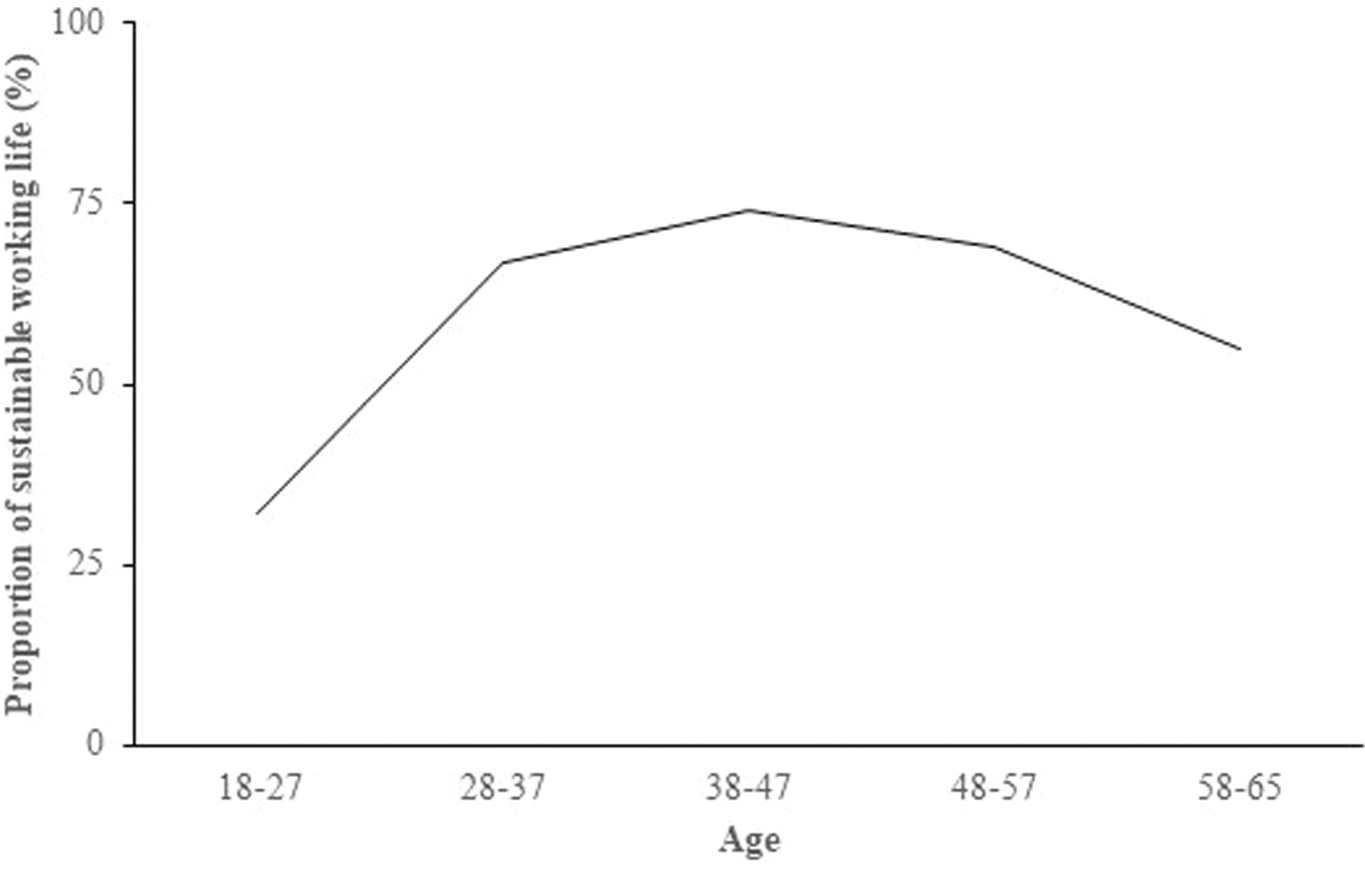
Mean proportion of sustainable working life across age.

The descriptives for the final sample are shown in Table 2. The youngest and the oldest age groups had the least (31% and 35%, respectively) of sustainable working life, whereas the highest level of sustainable working life was at the age group of 37–48 years (Fig 1). The proportion of women was slightly over 50% in all age groups. Twin correlations within each age group for MZ and DZ, and men and women are reported in Table 3.

**Table 2:** The demographics and mean with standard deviations (SD) for age at baseline of sustainable working life by age group.

Age group	Baseline year						
	Birthyear at baseline	Age at baseline	N total at baseline	Complete pairs at baseline^1^	Incomplete pairs at baseline	Women	Sustainable working life
	mean (range)	Mean (SD)	N = 51,079	n = 49,378	n = 1,701	%	%
18–27 years	1977 (1967–1990)	20 (3.0)	16,592	16,537	55	54	31
28–37 years	1962 (1957–1972)	33 (3.0)	8,470	8,339	131	52	68
38–47 years	1951 (1947–1963)	43 (3.0)	11,045	10,704	341	50	73
48–57 years	1942 (1937–1968)	52 (2.9)	10,406	9,936	470	53	65
58–65 years	1933 (1929–1960)	61 (2.3)	4,566	3,862	704	55	35
**Whole cohort**							
**Age group**	**mean (range)**	**Mean (SD)**	**N = 136,036**	**n = 129,884**	**n = 6,152**	**%**	**%**
18–27 years	1977 (1967–1990)	20 (3.0)	16,592	16,530	62	54	31
28–37 years	1971 (1957–1990)	24 (6.6)	24,488	23,864	624	53	68
38–47 years	1963 (1947–1982)	32 (9.8)	31,168	29,922	1246	52	74
48–57 years	1953 (1937–1972)	41 (10.0)	34,011	32,360	1651	52	69
58–65 years	1947 (1929–1962)	47 (8.5)	29,777	27,208	2569	53	51

^1^The final sample

**Table 3:** Within pair correlations by zygosity and sex in the final sample (n =  49,378 pairs) within each age group for sustainable working life.

Age groups	MZ	DZ	Women	Men
**Correlation**	**Correlation**	**Correlation**	**Correlation**
18–27 years	0.68	0.53	0.51	0.55
28–37 years	0.50	0.29	0.30	0.26
38–47 years	0.53	0.25	0.28	0.21
48–57 years	0.48	0.25	0.27	0.23
58–65 years	0.45	0.29	0.29	0.27

### Biometric analysis

**Fig 2:**
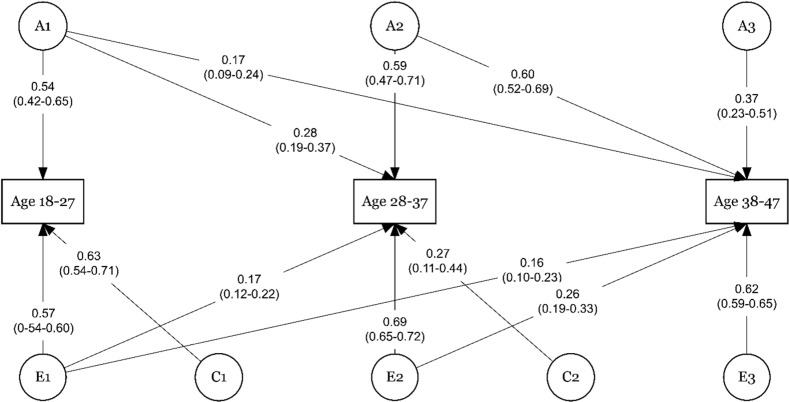
The trivariate Cholesky Decomposition of Sustainable working life at ages 18-27, 28-37, and 38-47 years of age with additive genetic (A), common environmental (C), and unique environmental (E) effects.

**Fig 3:**
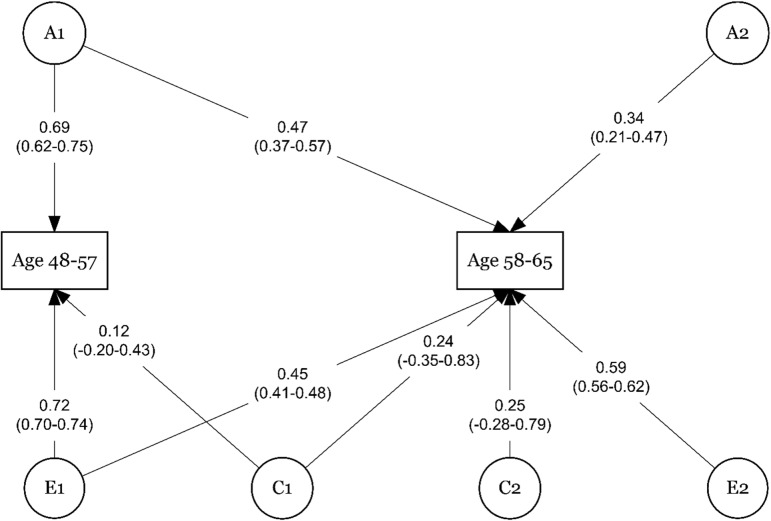
The bivariate Cholesky Decomposition of Sustainable working life at ages 48-57, and 58-65 years of age with additive genetic (A), common environmental (C), and unique environmental (E) effects.

We began the modeling by testing the assumption and ran the saturated model. Then a full ACE model including all five age groups as observed variables. This model yielded multiple paths estimates with zero leading us to drop paths. Since the cross-paths from younger age groups to the oldest age groups seemed to prevent the models from converging, we decided to model the sustainable working life across two sets of age groups. Thus, age groups 18–27, 28–37, and 38–47 were modeled together in a trivariate ACE-model, and age groups 48-57, and 58-65 were modeled together in a bivariate ACE-model. Comparisons of the tested models are presented in Tables 4 and 5. For both sets of age groups, the ACE model was the best and most parsimonious fit for data. The standardized, squared path estimates for the longitudinal ACE-models for age groups 18–47 are shown in Fig 2, and for age groups 48-65 in Fig 3.

**Table 4:** Model fitting statistics for age groups 18–47 years.

Model	EP	Δ Fit	Δ df	P-value	AIC	Δ AIC	-2lnL
Saturated	39				78,565		78,487
ACE^*^	**21**				**78,553**	**0**	78,511
ADE	21	66.1	0		78,619	66.1	78,577
AE	15	67.5	6	< 0.001	78,609	55.5	78,578
CE	15	207.4	6	< 0.001	78,748	195.4	78,718
E	9	2875	12	< 0.001	81,404	2,851	81,386
ACE without c1	18	63	3	< 0.001	78,610	57	78,574
ACE without c2	19	2.9	2	0.229	78,552	-1.1	78,514
ACE without c3	20	0	1	1.000	78,551	-2	78,511
ACE without c1 c2	16	71.5	5	< 0.001	78,615	61.5	78,582
ACE without c1 c3	17	63	4	< 0.001	78,608	55	78,574
ACE without c2 c3	18	2.9	3	0.399	78,550	-3.1	78,514
ACE without age 18–27 C	20	63	1	< 0.001	78,614	61	78,574
ACE without age 28–37 C	19	4.4	2	0.111	78,553	0.4	78,515
ACE without age 38–47 C	18	0.4	3	0.939	78,547	-5.6	78,511
ACE without age 18–27, 28–37 C	18	67.3	3	< 0.001	78,614	61.3	78,578
ACE without age 18–27, 38–47 C	17	63.4	4	< 0.001	78,608	55.4	78,574
ACE without age 28–37, 38–47 C	16	4.4	5	0.492	78,547	-5.6	78,515
**ACE without age 28-37 c1, age 38-47 C**	**17**	**0.9**	**4**	**0.930**	**78,546**	**-7.1**	**78,512**

Note. EP =  estimated parameters; Δ Fit =  change in Fit; Δ*df* =  change in degrees of freedom; AIC =  Akaike’s information criterion; Δ AIC =  change in Akaike’s information criterion; -2lnL =  − 2 times the log-likelihood. Best-fitting model is marked in bold.

* All subsequent models are compared to the ACE model.

**Table 5:** Model fitting statistics for age groups 48–65 years.

Model	EP	Δ Fit	Δ df	P	AIC	Δ AIC	-2lnL
Saturated	18				71,327		71,291
**ACE** ^*^	**11**				**71,326**	**0**	**71,304**
ADE	11	11.4	0		71,337	11.4	71,315
AE	8	11.4	3	0.010	71,331	5.4	71,315
CE	8	125.9	3	< 0.001	71,446	119.9	71,430
E	5	1402	6	< 0.001	72,716	1390	72,706
ACE without c1	9	0.9	2	0.650	71,323	-3.1	71,305
ACE without c2	10	0	1	0.829	71,324	-2	71,304
ACE without 48-57 C	10	0.9	1	0.353	71,325	-1.1	71,305
ACE without 58-65 C	9	11.2	2	0.004	71,333	7.2	71,315

Note. EP =  estimated parameters; Δ Fit =  change in Fit; Δ*df* =  change in degrees of freedom; AIC =  Akaike’s information criterion; Δ AIC =  change in Akaike’s information criterion; -2lnL =  -2 times the log likelihood. Best-fitting model is marked in bold.

* All subsequent models are compared to the ACE model.

As Figure 2 indicates, genetic influences for sustainable working life were 54% at ages 18–27 years, 59% at 28–37 years, and 37% at 38–47 years. Genetic influences originating from ages 18–27 years explained 28% of the variance in age intervals 28–37 years and 17% in 38–47 years, while the carry-on effect (i.e., effects spanning from earlier ages to later ones) of genetics from age group 28–37 years to 38–47 years was 60% (Fig 2). The role of a unique environment in sustainable working life was 57% at the age of 18–27 years, 69% at 28–37 years, and 62% at 38–47 years. Unique environment originating from ages 18–27 years explained 17% of variance in 28–37 years and 16% at 38–47 years. Variance explained by common environment was age-group specific for 18–27 years 63%, and 27% for 28–37 years of age whereas there was no effect of the common environment at the age group of 38–47 years.

Fig 3 shows that among the older age groups, the additive genetic component was 69% for ages 48–57 years and 34% for ages 58–65 years. Genetic effects originating from 48–57 years of age explained 47% of the genetic variance in 58–65 years. The unique environmental effect was 72% in 48–57 years of age and 59% in 58–65 years, while we also detected a carry-on effect of the unique environment in 48–57 years to 58–65 years (45%). Common environment (Fig 3) contributing to the variance in 48–57 years explained 12% of the variance in this age group and explained the variance in ages 58–65 years by 24%. The common environment, specific to the age group 58–65 years was 25%.

## Discussion

In this study based on a sample of 24,868 pairs, we aimed to investigate the age-specific genetic and environmental influences on sustainable working life from young adulthood until old-age pension. To the best of our knowledge, this is among the first population-based studies to focus on the stability and change in genetic and environmental influences on sustainable working life while earlier studies have focused on SA and/or DP [12–16]. We found that genetic influences for sustainable working life across the life course followed a two-peak distribution as genetic influences explained 54% of the variance at ages 18–27 years, 59% at 28–37 years, 37% at 38–47 years, 69% at 48–57 years, and 34% at 58–65 years. In addition, we detected a carry-on effect that increased towards older ages, with 28% of the variance in age intervals 28–37 years originating from ages 18–27 years and 17% in 38–37 years, from age group 28–37 years to 38–47 years was 60%, and from 48–57 years to 58–65 years 47%. Thus, these results indicate that there are genetic effects on sustainable working life, being both stable and changing in all age groups over time. Although there seem to be two peaks in genetic influence (i.e., ages 28–37, and 48–57), the potential mechanisms should be elaborated in further studies. One might speculate that these effects might be related to family formation, which also has been shown to carry a genetic effect [26] and affect sustainable working life, or perhaps traits related to fertility, i.e., having children [27] or healthy worker effect [28] may play a role.

However, the carry-on effects were larger in older compared to younger age groups. This deviates from the earlier findings in a Norwegian study focusing on DP over the life course that found rather stable genetic effects [12]. A potential explanation for the difference in findings is that the underlying disease and long-term processes of DP likely play a role, while a sustainable working life may reflect a more so-called “healthy worker” effect [29]. On the other hand, our results align with the knowledge that genetics play a role in educational attainment and socioeconomic status [30, 31], SA/DP based on period prevalence or incidence estimates [13,15], occupational choice[32], and the employment sector[33], which may influence and thus contribute to a sustainable working life.

The role of a unique environment in sustainable working life was overall moderate to large, being between 57% and 72%. However, the unique environment had minor carry-on effects in younger ages being 17% of variance originating from ages 18–27 years in 28–37 years, and 16% at 38–47 years, but 45% from 48–57 years to 58–65 years. A likely explanation might be that societal-level factors [34, 35] such as economics[36, 37], labor market, job availability, and welfare systems [38, 39]may affect sustainable working life differently across age groups. Another explanation might be social inequality which may differ between age groups and in which those lower educated are less healthy and more likely to have interruptions, i.e., having a non-sustainable working life [40].

The variance explained by common environmental factors was age group specific for 18–27 years being 63%, 27% for 28–37 years of age, and 25% for the oldest age group (58–65 years) whereas there was no effect of the common environment at the age group of 38–47 years. Thus, the common environment that is usually shared in childhood while living in the same family was strongest for the youngest ages to gradually decrease while people get older. The result is consistent with the common environmental effects of education [41], well-being [42], and longevity [43]. A likely mechanism is related to some shared lifestyle or circumstances at the early phases (such as childhood family, school, or neighborhood) of the life course.

All in all, although we can see both carry-on genetic and unique environmental effects since the youngest age group (18–27 years), the middle age groups, i.e., 28–57 years seem important for a sustainable working life. That might imply, given the relatively large role of the unique environment, that societal-level solutions such as social security, support for employment, or availability of jobs, but also workplace-level factors such as working conditions, workload, or social support might be relevant for sustainable working life, but also individual choices and events across working ages. Using longitudinal, good-quality, and comprehensive Swedish register data without loss to follow-up and recall biases were strengths while investigating genetic and environmental influences on sustainable working life using twin data. This register data enabled us to follow twins from their first steps in working life at the age of 18 years until the retirement age of 65 years. Furthermore, the large sample enabled the investigation of 10-year age groups to shed light on the different phases of the life course. Besides the Norwegian twin study of SA/DP [12], we are not aware of similar studies in this area of occupational epidemiology. Thus, this study adds also the findings of the earlier study based on partially the same dataset while assessing short- and long-term sustainable working life [2]. The register data might be considered a weakness as well since we lacked, e.g., information about workload, working environment, or choices that might potentially affect sustainable working life. Further studies should also address the potential effects of race, marriage, fertility, and region. Another weakness might be the fact that we needed to split the age groups due to problems with converging the model. This might imply two suggestions for further studies: even a larger sample size or a more detailed measure of sustainable working life could be applied to overcome these problems. Since Sweden is one of the Nordic countries with a welfare system to support interruptions of sustainable working life, our results are likely less generalizable to other countries outside of the Nordic.

To conclude, both stability and change in genetic effects explain sustainable working life across 10-year age groups from 18–27 years to 58–65 years. The unique environmental effects, i.e., circumstances not shared by the twins within a twin pair, played an important role, especially for the age groups between 28 and 57 years. This points to the direction of the importance of actions and measures the society and workplaces can provide to support individuals to stay in paid work and, in turn, sustainable working life.

## References

[1] Ropponen A, Wang M, Narusyte J, Silventoinen K, Bockerman P, Svedberg P, et al. Sustainable working life in a swedish twin cohort-A definition paper with sample overview. Int J Environ Res Public Health. 2021;18(11):5817. Epub 2021/06/03. doi: 10.3390/ijerph18115817 34071494 PMC8197988

[2] Ropponen A, Narusyte J, Wang M, Silventoinen K, Böckerman P, Svedberg P, et al. Genetic and environmental contributions to individual differences in sustainable working life-A Swedish twin cohort study. PLoS One. 2023;18(7):e0289074. Epub 2023/07/27. doi: 10.1371/journal.pone.0289074 ; PMCID: PMCPMC10374081 37498854 PMC10374081

[3] Ropponen A, Josefsson P, Böckerman P, Silventoinen K, Narusyte J, Wang M, et al. Sustainable working life patterns in a swedish twin cohort: age-related sequences of sickness absence, disability pension, unemployment, and premature death during working life. Int J Environ Res Public Health. 2022;19(17):10549. doi: 10.3390/ijerph191710549 36078264 PMC9517844

[4] ILO. Disability inclusion strategy and action plan 2014-17 a twin-track approach of mainstreaming and disability-specific actions. Geneva: International Labour Office; 2015.

[5] OECD. Sickness, Disability and Work: Breaking the Barriers. Paris: OECD Publishing; 2010.

[6] Collaborators GBDRF. Global, regional, and national comparative risk assessment of 79 behavioural, environmental and occupational, and metabolic risks or clusters of risks, 1990-2015: a systematic analysis for the global burden of disease study 2015. Lancet. 2016;388(10053):1659–724. Epub 2016/10/14. doi: 10.1016/S0140-6736(16)31679-8 ; PMCID: PMCPMC5388856 27733284 PMC5388856

[7] Commission E. Communication from the commission. Europe 2020. A strategy for smart, sustainable and inclusive growth Brussels: European Commission; 2010.

[8] Fisker J, Hjorthøj C, Hellström L, Mundy SS, Rosenberg NG, Eplov LF, et al. Predictors of return to work for people on sick leave with common mental disorders: a systematic review and meta-analysis. Int Arch Occup Environ Health. 2022;95(7):1–13. Epub 20220201. doi: 10.1007/s00420-021-01827-3 35106629

[9] Linder A, Gerdtham UG, Trygg N, Fritzell S, Saha S. Inequalities in the economic consequences of depression and anxiety in Europe: a systematic scoping review. Eur J Public Health. 2020;30(4):767–77. doi: 10.1093/eurpub/ckz127 ; PMCID: PMCPMC7445046 31302703 PMC7445046

[10] Harkko J, Virtanen M, Kouvonen A. Unemployment and work disability due to common mental disorders among young adults: selection or causation? Eur J Public Health. 2018;28(5):791–7. Epub 2018/03/08. doi: 10.1093/eurpub/cky024 29514230

[11] Eurofound. Sustainable working over the life course: concept paper. Publications Office of the European Union, Luxembourg: 2015.

[12] Seglem KB, Torvik FA, Roysamb E, Gjerde LC, Magnus P, Reichborn-Kjennerud T, et al. A life course study of genetic and environmental influences on work incapacity. Twin Res Hum Genet. 2020;23(1):16–22. Epub 2019/12/27. doi: 10.1017/thg.2019.116 31875789

[13] Narusyte J, Ropponen A, Silventoinen K, Alexanderson K, Kaprio J, Samuelsson A, et al. Genetic liability to disability pension in women and men: a prospective population-based twin study. PLoS One. 2011;6(8):e23143. Epub 2011/08/19. doi: 10.1371/journal.pone.0023143 ; PMCID: PMCPMC3151284 21850258 PMC3151284

[14] Svedberg P, Ropponen A, Alexanderson K, Lichtenstein P, Narusyte J. Genetic susceptibility to sickness absence is similar among women and men: findings from a Swedish twin cohort. Twin Res Hum Genet. 2012;15(5):642–8. Epub 2012/08/31. doi: 10.1017/thg.2012.47 22931554

[15] Gjerde LC, Knudsen GP, Czajkowski N, Gillespie N, Aggen SH, Roysamb E, et al. Genetic and environmental contributions to long-term sick leave and disability pension: a population-based study of young adult Norwegian twins. Twin Res Hum Genet. 2013;16(4):759–66. Epub 2013/06/08. doi: 10.1017/thg.2013.36 PubMed ; PMCID: PMCPMC3800163 23743022 PMC3800163

[16] Harkonmaki K, Silventoinen K, Levalahti E, Pitkaniemi J, Huunan-Seppala A, Klaukka T, et al. The genetic liability to disability retirement: a 30-year follow-up study of 24,000 Finnish twins. PLoS One. 2008;3(10):e3402. Epub 2008/10/17. doi: 10.1371/journal.pone.0003402 ; PMCID: PMCPMC2566596 18923678 PMC2566596

[17] Lichtenstein P, De Faire U, Floderus B, Svartengren M, Svedberg P, Pedersen NL, et al. The Swedish Twin Registry: a unique resource for clinical, epidemiological and genetic studies. J Intern Med. 2002;252(3):184–205. Epub 2002/09/25. doi: 10.1046/j.1365-2796.2002.01032.x 12270000

[18] Zagai U, Lichtenstein P, Pedersen NL, Magnusson PKE. The Swedish twin registry: content and management as a research infrastructure. Twin Res Hum Genet. 2019;22(6):672–80. Epub 2019/11/22. doi: 10.1017/thg.2019.99 31747977

[19] Ludvigsson JF, Svedberg P, Olen O, Bruze G, Neovius M. The longitudinal integrated database for health insurance and labour market studies (LISA) and its use in medical research. Eur J Epidemiol. 2019;34(4):423–37. Epub 2019/04/01. doi: 10.1007/s10654-019-00511-8 ; PMCID: PMCPMC6451717 30929112 PMC6451717

[20] Ropponen A, Wang M, Alaie I, Narusyte J, Svedberg P. Concurrent trajectories of residential region in relation to a sustainable working life among Swedish twins. Eur J Public Health. 2023;33(4):596–600. Epub 2023/04/09. doi: 10.1093/eurpub/ckad053 ; PMCID: PMCPMC10393480 37029917 PMC10393480

[21] Farrants K, Norberg J, Framke E, Rugulies R, Alexanderson K. Job demands and job control and future labor market situation: an 11-year prospective study of 2.2 million employees. J Occup Environ Med. 2020;62(6):403–11. doi: 10.1097/JOM.0000000000001859 32502083

[22] Farrants K, Head J, Framke E, Rugulies R, Alexanderson K. Associations between combinations of job demands and job control among 6,16,818 people aged 55-64 in paid work with their labour market status 11 years later: a prospective cohort study. Int Arch Occup Environ Health. 2022;95(1):169–85. Epub 20210607. doi: 10.1007/s00420-021-01717-8 ; PMCID: PMCPMC8755665 34097108 PMC8755665

[23] Boomsma D, Busjahn A, Peltonen L. Classical twin studies and beyond. Nat Rev Genet. 2002;3(11):872–82. Epub 2002/11/05. doi: 10.1038/nrg932 12415317

[24] Posthuma D, Beem AL, de Geus EJ, van Baal GC, von Hjelmborg JB, Iachine I, et al. Theory and practice in quantitative genetics. Twin Res. 2003;6(5):361–76. Epub 2003/11/20. doi: 10.1375/136905203770326367 14624720

[25] Loehlin JC. the cholesky approach: a cautionary note. Behav Genet. 1996;26(1):65–9. doi: 10.1007/bf02361160

[26] Gonggrijp BMA, Silventoinen K, Dolan CV, Boomsma DI, Kaprio J, Willemsen G. The mechanism of assortative mating for educational attainment: a study of Finnish and Dutch twins and their spouses. Front Genet. 2023;14:1150697. Epub 20230614. doi: 10.3389/fgene.2023.1150697 ; PMCID: PMCPMC10311485 37396041 PMC10311485

[27] Hart RJ. Physiological aspects of female fertility: role of the environment, modern lifestyle, and genetics. Physiol Rev. 2016;96(3):873–909. Epub 2016/06/03. doi: 10.1152/physrev.00023.2015 27252278

[28] Ropponen A, Wang M, Narusyte J, Silventoinen K, Böckerman P, Svedberg P. Sustainable working life in a swedish twin cohort-A definition paper with sample overview. Int J Environ Res Public Health. 2021;18(11):5817. Epub 2021/06/03. doi: 10.3390/ijerph18115817 ; PMCID: PMCPMC8197988 34071494 PMC8197988

[29] Eisen EA, Picciotto S, Robins JM. Healthy Worker Effect. Encyclopedia of Environmetrics, 2001.

[30] Marks GN. The contribution of genes and the environment to educational and socioeconomic attainments in Australia. Twin Res Hum Genet. 2017;20(4):281–9. Epub 2017/06/14. doi: 10.1017/thg.2017.32 28606205

[31] Roos JM, Nielsen F. Outrageous fortune or destiny? family influences on status achievement in the early life course. Soc Sci Res. 2019;80:30–50. Epub 2019/04/09. doi: 10.1016/j.ssresearch.2018.12.007 30955560

[32] Nicolaou N, Shane S. Entrepreneurship and occupational choice: genetic and environmental influences. J Econ Beh Organ. 2010;76(1):3–14. doi: 10.1016/j.jebo.2010.02.009

[33] Maczulskij T. Employment sector and pay gaps: Genetic and environmental influences. Lab Econ. 2013;23:89–96. doi: 10.1016/j.labeco.2013.04.009

[34] Drange I, Egeland C. Part-Time Work in the Nordic Region II: A research review on important reasons. Denmark: Nordic Council of Ministers; 2014.

[35] Commission E. Labour market policy. Expenditure and participants. Data 2015. Luxembourg: Publications Office of the European Union: European Union, 2017.

[36] Polvinen A, Laaksonen M, Gould R, Lahelma E, Leinonen T, Martikainen P. Socioeconomic differences in cause-specific disability retirement in finland, 1988 to 2009. J Occup Environ Med. 2016;58(8):840–5. Epub 2016/08/09. doi: 10.1097/JOM.0000000000000808 27500996

[37] Acciai F. The age pattern of social inequalities in health at older ages: are common measures of socio-economic status interchangeable? Public Health. 2018;157:135–41. Epub 2018/03/11. doi: 10.1016/j.puhe.2018.01.002 29524811

[38] Hytti H. Why are Swedes sick but Finns unemployed? Int J Soc Welfare. 2006;15(2):131–41. doi: 10.1111/j.1468-2397.2006.00412.x

[39] Myhr A, Haugan T, Lillefjell M, Halvorsen T. Non-completion of secondary education and early disability in Norway: geographic patterns, individual and community risks. BMC Public Health. 2018;18(1):682. Epub 2018/06/02. doi: 10.1186/s12889-018-5551-1 ; PMCID: PMCPMC5984305 29855297 PMC5984305

[40] Krokstad S, Johnsen R, Westin S. Social determinants of disability pension: a 10-year follow-up of 62 000 people in a Norwegian county population. Int J Epidemiol. 2002;31(6):1183–91. doi: 10.1093/ije/31.6.1183 12540720

[41] Silventoinen K, Jelenkovic A, Sund R, Latvala A, Honda C, Inui F, et al. Genetic and environmental variation in educational attainment: an individual-based analysis of 28 twin cohorts. Sci Rep. 2020;10(1):12681. Epub 2020/07/31. doi: 10.1038/s41598-020-69526-6 ; PMCID: PMCPMC7391756 32728164 PMC7391756

[42] Bartels M. Genetics of wellbeing and its components satisfaction with life, happiness, and quality of life: a review and meta-analysis of heritability studies. Behav Genet. 2015;45(2):137–56. doi: 10.1007/s10519-015-9713-y 25715755 PMC4346667

[43] Caruso C, Ligotti ME, Accardi G, Aiello A, Duro G, Galimberti D, et al. How important are genes to achieve longevity? Int J Mol Sci. 2022;23(10):5635. Epub2022/05/29. doi: 10.3390/ijms23105635 ; PMCID: PMCPMC9145989 35628444 PMC9145989

